# Cell Painting and machine learning for mechanistic profiling of hepatotoxic botanicals and their constituents

**DOI:** 10.64898/2026.10.05.756246

**Published:** 2026-10-12

**Authors:** Guillaume Ollitrault, Constance A. Mitchell, Stephen S. Ferguson, Amy L. Roe, Brandon White, Katherine Titterton, Jessica Ewald

## Abstract

**BACKGROUND:**

Botanicals are widely consumed as herbal remedies and plant-derived products, but their complex and variable chemical composition presents major challenges for safety assessment. Although hepatotoxicity has been reported for several botanicals, the constituent compounds responsible and the underlying mechanisms often remain poorly characterised.

**OBJECTIVES:**

We aimed to determine whether Cell Painting could identify candidate constituent drivers of botanical bioactivity and generate hypotheses about potential mechanisms of action in primary human hepatocytes.

**METHODS:**

We profiled 13 botanical extracts and 85 known constituents across multiple concentrations using Cell Painting alongside assays of metabolic activity and membrane integrity. To interpret morphological phenotypes, we trained machine-learning models using a Cell Painting reference atlas of >1,000 chemicals linked to ToxCast/Tox21 molecular and cellular bioactivity annotations.

**RESULTS:**

Cell Painting detected bioactivity in 11/13 botanical extracts and 46/85 constituents, compared with 9/13 extracts and 40/85 constituents in the cell-health assays, and generally detected activity at lower concentrations. For 9/13 extracts, at least one constituent was active at a lower concentration than the corresponding botanical extract, supporting the contribution of a subset of potent constituents to mixture-level responses. Aristolochia and goldenseal were the most potent extracts across Cell Painting and cell-health assays and contained highly active constituents. Reference-atlas models generated specific mechanistic hypotheses, including cytotoxicity-, endocrine-, and genotoxicity-associated responses for several botanicals and constituents.

**CONCLUSION:**

These results support Cell Painting combined with machine learning as a sensitive approach for characterising cellular responses to botanicals, prioritising candidate constituent drivers of hepatotoxicity-associated phenotypes, and generating testable hypotheses about potential mechanisms of action.

